# Research agenda for black prostate health: issues, challenges and future directions

**DOI:** 10.1186/1750-9378-6-S1-A1

**Published:** 2011-08-11

**Authors:** Camille CR Ragin

**Affiliations:** 1 Cancer Prevention and Control Program, Fox Chase Cancer Center, USA

## Background

A disproportionate burden of prostate cancer among Black men continues to be observed worldwide. Between 1975 and 2007 the age adjusted incidence of prostate cancer in African-American men was 235 per 100,000 compared to White men (149 per 100,000). Similarly a racial disparity in mortality rates is observed in the United States (White men: 29.97 per 100,000 vs. Black men: 66.49 per 100,000). Globally, prostate cancer incidence and mortality rates also vary geographically. According to the World Health Organization, higher incidence and mortality rates are observed in more developed regions compared to less developed regions of the world. Prostate cancer continues to be a major public health issue and efforts to address the global prostate cancer disparities in Black males are met with many challenges, specifically in developing nations.

## Methods

During a Lunch “N” Dialogue at the Global Prostate Cancer conference held in August 27-29, 2010, challenges and future directions for the Research Agenda for Black Prostate Health were addressed.

## Results

Some of the challenges identified were competitive funding opportunities, limited funding available to non-profit community-based organizations, limited resources for research and limited career development and mentorship opportunities. In developing regions, such as Africa and the Caribbean, low screening rates prohibit the estimation of baseline levels of prostate cancer incidence. Furthermore, there is little known about the level of knowledge and attitudes among practitioners with regard to prostate cancer screening. An engaging discussion yielded suggestions of the future directions that might help to address some of these challenges. There was a clear expression of a need for the translation of research to the community by increasing the educational messages to the community and increasing educational interventions at the community level through the utilization of culturally-specific research methodologies for the Black population.

## Conclusion

Further assessments of knowledge and attitudes of practitioner screening in developing countries as well as continuing education of physicians in order to promote their engagement and involvement in research were also suggested. To address the majority of these issues, the increased availability of funding sources for non-profit organizations involved in community based research is a priority. Most importantly, there should be establishment and sustainment of a network of global collaborators that will seek to collectively address some of the challenges that are faced in developing regions.

## Acknowledgements

Publication of this article was funded in part by the University of Florida Open-Access Publishing Fund.

